# Association of Light-Intensity Physical Activity With Mortality in the Older Population: A Nationwide Cohort Study

**DOI:** 10.3389/fcvm.2022.859277

**Published:** 2022-04-22

**Authors:** Juntae Kim, Pil-Sung Yang, Byoung-Eun Park, Tae Soo Kang, Seong-Hoon Lim, Sungsoo Cho, Su-Yeon Lee, Young Hak Chung, Myung-Yong Lee, Dongmin Kim, Boyoung Joung

**Affiliations:** ^1^Division of Cardiology, Department of Internal Medicine, College of Medicine, Dankook University, Cheonan-si, South Korea; ^2^Department of Cardiology, CHA Bundang Medical Center, CHA University, Seongnam-si, South Korea; ^3^Division of Cardiology, Department of Internal Medicine, Yonsei University College of Medicine, Seoul, South Korea

**Keywords:** sport cardiology, exercise, light-intensity physical activity, elderly, all-cause mortality, cardiovascular mortality

## Abstract

**Background:**

There is a paucity of information about mortality related to light-intensity physical activity (LPA) in the older population. We examine the associations between physical activity and mortality, focusing on the effect of light-intensity physical activity and the dose-response relationship between physical activity and mortality.

**Methods:**

We analyzed a total of 58,537 participants aged ≥ 65 years (mean age, 73.9 ± 5.8 years; male, 36.0%) in the Korean National Health Insurance Service database between 2009 and 2012. The Date of the end of follow-up was December 31, 2013. Individuals were divided into four categories according to physical activity intensity: totally sedentary (43.3%), LPA only (35.8%), LPA and moderate- to vigorous-intensity physical activity (MVPA) (16.3%), MVPA only (4.5%). Physical activity was quantified using standardized self-reported questionnaires which composed of the duration and frequency of physical activity.

**Results:**

During a mean follow-up of 39.6 ± 14.0 months, 5,651 (9.7%) deaths occurred. Compared with totally sedentary individuals, those in the LPA only, LPA and MVPA, and MVPA only groups showed 26% [hazard ratio (HR) 0.74, 95% confidence interval (CI) 0.68–0.82], 27% (HR 0.73, 95% CI 0.63–0.84), and 34% (HR 0.66, 95% CI 0.54–0.79) lower all-cause mortality risk, showing an inverse relationship between physical activity intensity and mortality risk. In contrast, the LPA only, LPA and MVPA, and MVPA only groups represented a stronger inverse association with CV mortality (LPA: HR 0.76, 95% CI 0.62–0.92; LPA with MVPA: HR 0.74, 95% CI 0.55–0.999; MVPA, HR 0.57, 95% CI 0.37–0.87). Among participants performing LPA alone, participants performing less than the recommended dose of physical activity had lower all-cause mortality than those with sedentary activity (1–249 MET-min/week: HR 0.74, 95% CI 0.67–0.82, 250–499 MET-min/week: HR 0.65, 95% CI 0.59–0.72).

**Conclusion:**

Physical activity, even low doses of LPA, was associated with reduced mortality risk in the elderly population. This study may motivate sedentary individuals to engage in any physical activity for mortality benefits.

## Introduction

Physical activity is associated with a reduced risk of vascular, non-vascular disease, and mortality (1). Recent physical activity guidelines recommend at least 150–300 min per week of moderate-intensity physical activity (MPA) or 75–150 min per week of vigorous-intensity physical activity (VPA), which is equivalent to 500–999 metabolic equivalent task (MET)-min/week, in elderly (aged ≥ 65 years) (2, 3). Achieving >150 min/week moderate-intensity aerobic exercise is associated with a lower risk of morbidity, mortality, disability, and frailty compared with being sedentary (4, 5). However, most guidelines of physical activity are similar in middle-aged adults and older adults (3). It is estimated that >60% of elderly adults could not achieve 150 min per week of moderate- to vigorous-intensity physical activity (MVPA) (6). They have difficulty engaging in exercise due to their health condition. Also, the insufficiency of knowledge of the association between physical activity and health benefits and lack of physician advice to encourage exercise were barriers to exercise (6). Recently, the benefits of physical activity at doses below the current guideline-recommended level were reported. Wen et al. (7) reported health benefits in individuals who engaged in moderate-intensity physical activity at half the recommended amount. Hupin et al. reported a low dose of MVPA below recommended level reduced mortality by 22% in elderly adults. Mortality was further reduced in those who engaged in a higher dose of physical activity in a dose-dependent manner (8). However, whether light-intensity physical activity (LPA) can reduce mortality in the older population has not been revealed. To investigate this association, we analyzed the association between physical activity and mortality in older adults in a nationwide cohort. We focused on the dose-response relationship between LPA and mortality.

## Materials and Methods

### Study Population

Data were collected from the National Health Insurance Service of Korea (NHIS)-Senior database, which included data on 558,147 individuals recruited by the 10% simple random sampling method from a total of 5.5 million subjects aged ≥ 60 years in the National Health Information Database (9, 10). The NHIS-Senior database covered the following parameters: sociodemographic and socioeconomic information, health check-up examinations, insurance status, and records of participants’ medical histories. This study was approved by the Institutional Review Board of the Yonsei University Health System (4-2021-0850).

**FIGURE 1:**
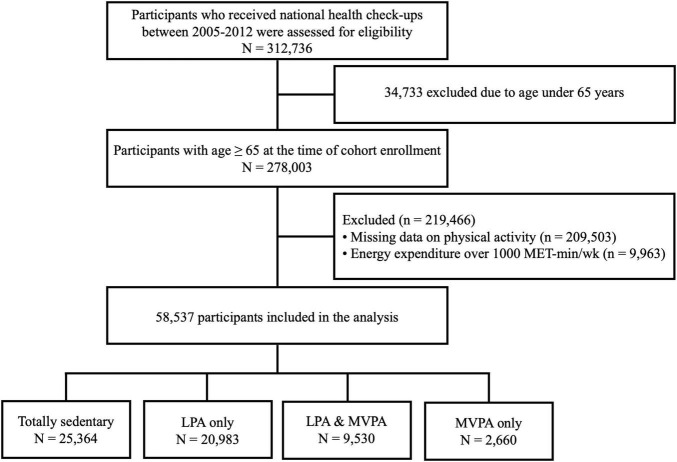
Summary of the statistical analysis design. LPA, light intensity physical activity; MVPA, moderate- to vigorous-intensity physical activity; MET, metabolic equivalent of task.

From the Korean NHIS-Senior database, 278,003 participants aged ≥ 65 years who had available health check-up data between 2009 and 2012 were enrolled in this study. We excluded those with missing information on physical activity (*n* = 209,503). Also, those who achieved energy expenditure exceeding the guideline target range (>1,000 MET min/week) (*n* = 9,963) were excluded to evaluate the dose-response of physical activity below the recommended dose. Finally, a total of 58,537 subjects were included in the study and followed up until December 2013 (Figure 1).

### Physical Activity Level Assessment

The leisure-time physical activity level was quantified using standardized self-reported questionnaires using a 7-day recall method (11). The survey was composed of three questions that addressed the usual frequency (days per week) of (i) VPA for at least 20 min, (ii) MPA for at least 30 min, and (iii) LPA for at least 30 min. VPA was defined as intense exercise that caused severe shortness of breath, such as running and bicycling at high speed. MPA was defined as exercise that caused mild shortness of breath, such as brisk walking and bicycling at the usual speed. LPA was defined as walking at a slow or leisurely pace.

Ratings of 3.0, 4.0, and 8.0 METs were assigned for LPA, MPA, and VPA, respectively (12). Physical activity-related energy expenditure (MET-min/week) was calculated by summing the product of frequency, intensity, and duration. The total energy expenditure level was stratified into 0, <250, 250–499, and 500–1,000 in an explorative manner. Totally sedentary group was defined as individuals without any leisure-time physical activity. The participants were categorized according to physical activity intensity into LPA only, LPA and MVPA, and MVPA only groups.

### Covariates

Sociodemographic variables included age, gender, and economic status. The baseline economic status was determined based on the relative economic levels categorized into 11 levels ranging from group 0 (lowest) to group 10 (highest) according to their health insurance premiums paid, which directly reflect each individual’s income level. Baseline comorbidities were verified using the International Classification of Disease-10 (ICD-10) codes and prescription medication before the index data (Supplementary Table 1). To improve diagnostic accuracy, comorbidities were identified when the condition was a discharge diagnosis or was recorded at least twice in an outpatient setting, similar to our previous studies (10, 12–20).

### Outcomes

The primary outcome was all-cause mortality. The date of death and ICD-10 codes were verified from the National Population Registry of the Korea National Statistical Office using unique personal identification numbers (10, 12–20). NHIS and the National Statistical Office are national agencies serving all residents in the Republic of Korea, providing a complete event check. We investigated cause-specific mortality based on causes of death because exercise can be associated with cardiovascular (CV)-related and non-CV-related mortality. We defined CV death as death from diseases of the circulatory system (ICD codes I00–I78). The follow-up period was defined as the time from the index date that participants registered in this cohort to death or the end of the study period, whichever came first.

### Statistical Analyses

Descriptive statistics were used to analyze baseline characteristics and comorbidities. Categorical variables are expressed as frequencies (percentages). Continuous variables were reported as means ± standard deviations or medians with interquartile ranges. Categorical variables were compared using the Pearson chi-square test, and continuous variables were compared using one-way analysis of variance or the Kruskal-Wallis test, as appropriate.

The incidence rates of outcomes were calculated by dividing the number of events by person-time at risk and presented as the rate per 1,000 person-years. We analyzed the hazard ratios (HRs) and 95% confidence intervals (CIs) for mortality according to physical activity level. Multivariable Cox regression models were constructed with adjustment for age, sex, income level, residential area (urban or non-urban), body mass index, hypertension, diabetes mellitus, dyslipidemia, smoking, alcohol intake, chronic kidney disease, chronic obstructive lung disease, chronic liver disease, malignancy, cardiovascular medications (aspirin, P_2_Y_12_ inhibitor, statin, anticoagulant, beta-blocker, angiotensin converting enzyme inhibitor or angiotensin receptor blocker, calcium channel blocker, digoxin, and diuretics) and Charlson comorbidity index. Categorical measures of physical activity intensity and energy expenditure level were treated as an ordered value in Cox regression analysis to evaluate *P* value for linear trend. Restricted cubic spline curves were used to examine the effects of continuous values of physical activity (0 MET-min/week as reference) on death. Restricted cubic splines were fitted with three knots by treating the amount of physical activity as a continuous variable using the “rms” package.

We conducted subgroup analyses for the primary outcome stratified by age, sex, body mass index, and other baseline comorbidities. For sensitivity analysis, we performed separate analyses in those who did not perform any activity exceeding LPA.

All tests were two-tailed, with a *p*-value of <0.05 considered significant. Statistical analyses were conducted using R programming version 4.1.0 (The R Foundation for Statistical Computing, Vienna, Austria).

## Results

### Baseline Characteristics

In total, 58,537 participants (men 36.0%) with a mean age of 73.9 ± 5.8 years were included in the analysis. Baseline characteristics according to physical activity intensity are summarized in Table 1. With regard to physical activity intensity, 43.3, 35.8, 16.3, and 4.5% of participants were included in the totally sedentary, LPA only, LPA and MVPA, and MVPA only groups, respectively. Of the total participants, 46,347 (79.2%) did not perform any activity beyond LPA. The distribution of exercise dose according to physical activity intensity is presented in Supplementary Figure 1. Participants performing physical activity tended to be younger, male-predominant, less likely to have comorbidities (particularly hypertension, chronic obstructive pulmonary disease (COPD), chronic kidney disease, and osteoporosis), and less affected by gait disturbances during the Timed Up and Go Test than totally sedentary participants. There were no significant differences in Activities of Daily Living scale, which represents the functional status of older adults.

**TABLE 1:** Baseline characteristics.

	Totally sedentary (*N* = 25,364)	LPA only (*N* = 20,983)	LPA & MVPA (*N* = 9,530)	MVPA only (*N* = 2,660)
Age, mean (SD)	74.9 ± 6.4	73.4 ± 5.3	72.7 ± 5.0	72.1 ± 4.8
Male	8,224 (32.4%)	7,541 (35.9%)	4,012 (42.1%)	1,280 (48.1%)
BMI, mean (SD)	23.5 ± 3.6	23.8 ± 3.4	23.9 ± 3.2	24.1 ± 3.2
**Economic status**				
Low	8,321 (32.8%)	6,387 (30.4%)	2,814 (29.5%)	746 (28.0%)
Middle	5,721 (22.6%)	4,617 (22.0%)	2,014 (21.1%)	611 (23.0%)
High	11,322 (44.6%)	9,979 (47.6%)	4,702 (49.3%)	1,303 (49.0%)
Systolic blood pressure (mmHg), mean (SD)	131.2 ± 17.5	131.6 ± 17.0	130.9 ± 16.8	131.0 ± 16.4
Diastolic blood pressure (mmHg), mean (SD)	78.3 ± 10.7	78.3 ± 10.4	78.3 ± 10.2	77.9 ± 10.0
Hypertension	15,888 (62.6%)	12,836 (61.2%)	5,716 (60.0%)	1,535 (57.7%)
Diabetes mellitus	5,315 (21.0%)	4,648 (22.2%)	1,969 (20.7%)	580 (21.8%)
Dyslipidemia	12,496 (49.3%)	10,977 (52.3%)	5,020 (52.7%)	1,408 (52.9%)
CKD or ESRD	7,009 (27.7%)	5,036 (24.0%)	2,110 (22.2%)	568 (21.4%)
COPD	3,559 (14.0%)	2,558 (12.2%)	1,120 (11.8%)	285 (10.7%)
Liver disease	7574 (29.9%)	6,455 (30.8%)	2,922 (30.7%)	795 (29.9%)
Any malignancy	4,018 (15.8%)	3,418 (16.3%)	1,550 (16.3%)	454 (17.1%)
Current smoker	2,776 (11.0%)	2,466 (11.8%)	1,170 (12.4%)	325 (12.3%)
Current alcohol drinker	1,877 (7.4%)	1,841 (8.8%)	927 (9.8%)	327 (12.4%)
Charlson comorbidity index, mean (SD)	3.3 ± 2.8	3.2 ± 2.7	3.0 ± 2.6	2.9 ± 2.6
**Total physical activity, METs**				
0	25,364 (100.0%)	0	0	0
1-500	0	11,980 (57.1%)	3,318 (34.8%)	1,497 (56.3%)
500-1000	0	9,003 (42.9%)	6,212 (65.2%)	1,163 (43.7%)
Depressive mood	1,507 (54.0%)	1,589 (54.0%)	850 (50.9%)	284 (55.3%)
**Lower extremity function**				
Gait disturbance during TUG†	96 (3.5%)	80 (2.7%)	35 (2.1%)	5 (1.0%)
Time taken in TUG (sec)^†^, median [Q1, Q3]	9.0 [7.0,10.0]	9.0 [7.0,10.0]	9.0 [7.0,10.0]	8.0 [7.0,10.0]
**Cognitive function**				
Positive rate in KDSQ^‡^	649 (23.2%)	669 (22.7%)	367 (21.9%)	116 (22.6%)
KDSQ score^‡^, median [Q1, Q3]	1.0 [0.0, 3.0]	1.0 [0.0, 3.0]	1.0 [0.0, 3.0]	1.0 [0.0, 3.0]
ADL scale^§^, median [Q1, Q3]	6.0 [6.0, 7.0]	6.0 [6.0, 7.0]	6.0 [6.0, 7.0]	6.0 [6.0, 7.0]
**Medication**				
Aspirin	9,141 (36.0%)	7,572 (36.1%)	3,473 (36.4%)	941 (35.4%)
P_2_Y_12_ inhibitor	2,377 (9.4%)	1,833 (8.7%)	754 (7.9%)	221 (8.3%)
Statin	5,956 (23.5%)	5,590 (26.6%)	2,498 (26.2%)	694 (26.1%)
Anticoagulant	380 (1.5%)	345 (1.6%)	118 (1.2%)	40 (1.5%)
ACE inhibitor or ARB	9,184 (36.2%)	7,592 (36.2%)	3,397 (35.6%)	930 (35.0%)
Beta-blocker	7,091 (28.0%)	5,856 (27.9%)	2,603 (27.3%)	669 (25.2%)
Calcium channel blocker				
DHP	10,997 (43.4%)	8,884 (42.3%)	3,877 (40.7%)	1,067 (40.1%)
Non-DHP	1,214 (4.8%)	1,018 (4.9%)	452 (4.7%)	116 (4.4%)
Digoxin	812 (3.2%)	575 (2.7%)	218 (2.3%)	60 (2.3%)
Diuretics	10,299 (40.6%)	8,137 (38.8%)	3,521 (36.9%)	940 (35.3%)

*Values are expressed in n (%), mean ± SD (standard deviation), or median [Q1, Q3].*

*ADL, activities of daily living; BMI, body mass index; CKD, chronic kidney disease; COPD, chronic obstructive pulmonary disease; ESRD, end-stage renal disease; KDSQ, Korean Dementia Screening Questionnaires; LPA, light intensity physical activity; MET, metabolic equivalent of task; MVPA, moderate- to vigorous-intensity physical activity; TUG, timed up and go test.*

*^†^TUG test measures the time that a person takes to rise from a chair, walk 3 m, turn around, walk back to the chair, and sit down. The more time taken indicates the poor physical function and balance.*

*^‡^KDSQ including five items. Each item on the KDSQ is scored from 0 to 2, with a higher score indicating poorer function and a greater frequency. KDSQ score ≥ 4 indicates positive. ^§^ A measurement of routine activities people do every day without assistance, which include eating, bathing, getting dressed, toileting, mobility, and continence. Higher scores indicate better and independent physical performance.*

*^§^ A measurement of routine activities people do every day without assistance, which include eating, bathing, getting dressed, toileting, mobility, and continence. Higher scores indicate better and independent physical performance.*

### Physical Activity Intensity, All-Cause Mortality, Cardiovascular Mortality, and Non-cardiovascular Mortality

During a mean follow-up of 39.6 ± 14.0 months, 5,651 (9.7%) deaths occurred. The overall incidence of mortality during follow-up was 29.3 per 1,000 person-years. When stratified by physical activity intensity, the incidence was 40.5, 22.6, 19.0, and 16.2 per 1,000 person-years in the totally sedentary, LPA only, LPA and MVPA, and MVPA only groups, respectively (Table 2). Compared with the totally sedentary group, the LPA only (HR 0.74, 95% CI 0.68–0.82), LPA and MVPA (HR 0.73, 95% CI 0.63–0.84), and MVPA only groups (HR 0.66, 95% CI 0.54–0.79) were associated with a lower risk of mortality.

**TABLE 2:** Incidence and hazard ratio with 95% confidence intervals for all-cause death, cardiovascular (CV) cause death, and non-CV-cause death according to physical activity intensity.

Group	Total Patient, *n*	Event, *n*	Crude incidence per 1,000 Patient-Years	Absolute reduction in event rate (95% CI)	Hazard ratio (95% CI)*	p-Value	p for trend^†^
**All-cause mortality**							<0.001
Totally sedentary	25,364	3,316	40.5		1.00 (reference)		
LPA only	20,983	1,572	22.6	17.9 (16.2–19.6)	0.74 (0.68–0.82)	<0.001	
LPA and MVPA	9,530	613	19.0	21.5 (19.4–23.5)	0.73 (0.63–0.84)	<0.001	
MVPA only	2,660	150	16.2	24.2 (21.3–27.1)	0.66 (0.54–0.79)	<0.001	
**CV-cause mortality**							0.006
Totally sedentary	25,364	880	10.7		1.00 (reference)		
LPA only	20,983	361	5.2	5.6 (4.7–6.4)	0.76 (0.62–0.92)	0.006	
LPA and MVPA	9,530	129	4.0	6.7 (5.8–7.7)	0.74 (0.55–0.999)	0.049	
MVPA only	2,660	27	2.9	7.8 (6.5–9.1)	0.57 (0.37–0.87)	0.009	
**Non-CV-cause mortality**							<0.001
Totally sedentary	25,364	2,436	29.7		1.00 (reference)		
LPA only	20,983	1,211	17.4	12.3 (10.8–13.9)	0.74 (0.66–0.83)	<0.001	
LPA and MVPA	9,530	484	15.0	14.7 (13.0–16.5)	0.73 (0.62–0.86)	<0.001	
MVPA only	2,660	123	13.3	16.4 (13.8–19.0)	0.68 (0.55–0.84)	<0.001	

*CI, confidence interval; LPA, light intensity physical activity; MVPA, moderate- to vigorous-intensity physical activity.*

*Adjusted for age, sex, income level, residential area (urban or non-urban), body mass index, hypertension, diabetes mellitus, dyslipidemia, smoking, alcohol intake, chronic kidney disease, chronic obstructive lung disease, liver disease, malignancy, cardiovascular medications (aspirin, P_2_Y_12_ inhibitor, statin, anticoagulant, beta-blocker, angiotensin converting enzyme inhibitor or angiotensin receptor blocker, calcium channel blocker, digoxin, diuretics), Charlson comorbidity index and energy expenditure.

*^†^Categorical measures of physical activity intensity were treated as an ordered value.*

The incidence of CV mortality was 10.7, 5.2, 4.0, and 2.9 per 1,000 person-years in the totally sedentary, LPA only, LPA and MVPA, and MVPA only groups, respectively. Compared with the totally sedentary group, the LPA only (HR 0.76, 95% CI 0.62–0.92), LPA and MVPA (HR 0.74, 95% CI 0.55–0.999), and MVPA only groups (HR 0.57, 95% CI 0.37–0.87) were associated with a lower risk of mortality (Table 2).

The incidence of non-CV mortality was 29.7, 17.4, 15.0, and 13.3 per 1,000 person-years in the totally sedentary, LPA only, LPA and MVPA, and MVPA only groups, respectively. Compared with the totally sedentary group, the LPA only (HR 0.74, 95% CI 0.66–0.83), LPA and MVPA (HR 0.73, 95% CI 0.62–0.86), and MVPA only groups (HR 0.68, 95% CI 0.55–0.84) were associated with a lower risk of mortality (Table 2).

The cumulative incidence of all-cause mortality, CV mortality, and non-CV mortality showed progressively lower trends with increasing physical activity intensity (Supplementary Figure 2).

### Physical Activity Dose and Mortality

**FIGURE 2:**
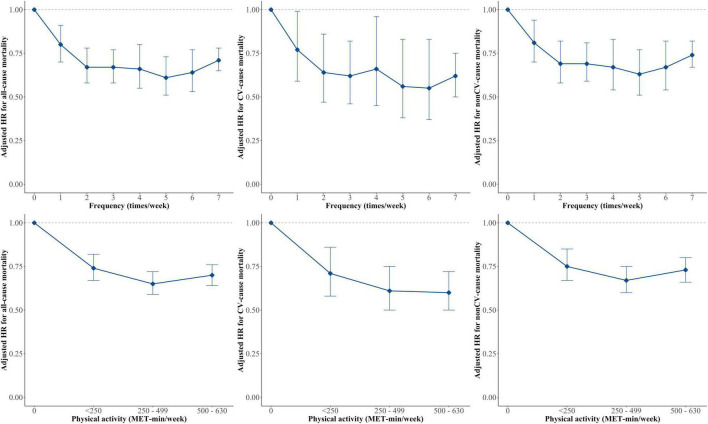
Hazard ratios (HRs) of All-cause, CV, and non-CV-mortality by exercise frequency, and physical activity level in those who did not perform any activity beyond LPA. Multivariable Cox regression models were constructed with adjustment for age, sex, income level, residential area (urban or non-urban), body mass index, hypertension, diabetes mellitus, dyslipidemia, smoking, alcohol intake, chronic kidney disease, chronic obstructive lung disease, liver disease, malignancy, cardiovascular medications (aspirin, P_2_Y_12_ inhibitor, statin, anticoagulant, beta-blocker, angiotensin converting enzyme inhibitor or angiotensin receptor blocker, calcium channel blocker, digoxin, diuretics) and Charlson comorbidity index. CV, cardiovascular; MPA, moderate-intensity physical activity; MET, metabolic equivalent of task.

Among the 58,537 participants, 46,347 (79.2%) participants, including 20,983 with LPA, did not perform any activity beyond LPA. In the LPA only group, 4,971 (10.7%), 7,009 (15.1%), and 9,003 (19.4%) participants performed 1–249, 250–499, and 500–1,000 MET-min/week of LPA, respectively. The cumulative incidence of mortality was lower in the group with 250–499 MET-min/week of LPA (Supplementary Figure 3). Compared with those in the totally sedentary group, participants with 1–249 MET-min/week of LPA (HR 0.74, 95% CI 0.67–0.82), 250–499 MET-min/week (HR 0.65, 95% CI 0.59–0.72), and 500–1,000 MET-min/week of LPA (HR 0.70, 95% CI 0.64–0.76) showed a lower risk of mortality (Table 3). Similar trends were observed in the MVPA group (Supplementary Figure 4). Even once a week of LPA was associated with significantly lower risks of all-cause mortality than the totally sedentary group (Figure 2).

**TABLE 3:** Incidence and hazard ratio with 95% confidence intervals for all-cause death, CV cause death, and non-CV-cause death in those who did not perform any activity beyond LPA, according to energy expenditure (MET-min/week).

	Total patient, *n*	Event, *n*	Crude incidence per 1,000 patient-years	Absolute reduction in event rate (95% CI)	Hazard ratio (95% CI)*	p-value	p for trend^†^
**All-cause mortality**							<0.001
Totally sedentary	25,364	3,316	40.5		1.00 (reference)		
<250 MET-min/week	4,971	449	26.6	13.9 (11.1–16.7)	0.74 (0.67–0.82)	<0.001	
250–500 MET-min/week	7,009	454	19.6	20.9 (18.6–23.1)	0.65 (0.59–0.72)	<0.001	
500–1,000 MET-min/week	9,003	669	22.6	17.9 (15.7–20.0)	0.70 (0.64–0.76)	<0.001	
**CV-cause mortality**							<0.001
Totally sedentary	25,364	880	10.7		1.00 (reference)		
<250 MET-min/week	4,971	111	6.6	4.2 (2.8–5.6)	0.71 (0.58–0.86)	0.001	
250–500 MET-min/week	7,009	107	4.6	6.1 (5.0–7.2)	0.61 (0.50–0.75)	<0.001	
500–1,000 MET-min/week	9,003	143	4.8	5.9 (4.8–7.0)	0.60 (0.50–0.72)	<0.001	
**Non-CV-cause mortality**							<0.001
Totally sedentary	25,364	2,436	29.7		1.00 (reference)		
<250 MET-min/week	4,971	338	20.0	9.7 (7.3–12.2)	0.75 (0.67–0.85)	<0.001	
250–500 MET-min/week	7,009	347	15.0	14.7 (12.8–16.7)	0.67 (0.60–0.75)	<0.001	
500–1,000 MET-min/week	9,003	526	17.8	12.0 (10.1–13.9)	0.73 (0.66–0.80)	<0.001	

*CI, confidence interval; LPA, light intensity physical activity; MVPA, moderate- to vigorous-intensity physical activity.*

*Adjusted for age, sex, income level, residential area (urban or non-urban), body mass index, hypertension, diabetes mellitus, dyslipidemia, smoking, alcohol intake, chronic kidney disease, chronic obstructive lung disease, liver disease, malignancy, cardiovascular medications (aspirin, P_2_Y_12_ inhibitor, statin, anticoagulant, beta-blocker, angiotensin converting enzyme inhibitor or angiotensin receptor blocker, calcium channel blocker, digoxin, diuretics) and Charlson comorbidity index.

*^†^Categorical measures of total energy expenditure level were treated as an ordered value.*

### Non-linear Relationship Between Physical Activity and All-Cause Mortality

**FIGURE 3:**
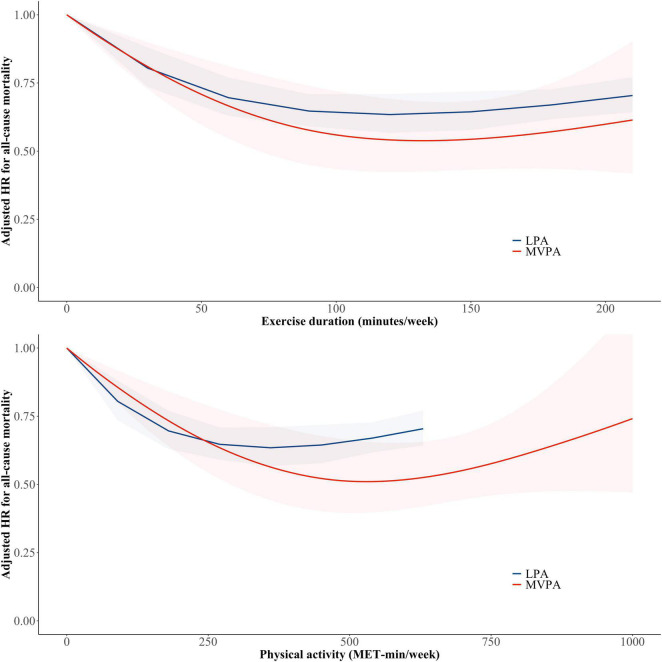
Non-linear relationship between physical activity and all-cause mortality risk according to physical activity intensity. Restricted cubic spline curves were constructed with regard to physical activity treated as a continuous variable. The red and blue lines and shades indicate adjusted hazard ratio and 95% confidence intervals for subjects with light-intensity physical activity and moderate to vigorous physical activity, respectively. LPA, light-intensity physical activity; MET, metabolic equivalent of task; MVPA, moderate- to vigorous-intensity physical activity.

Figure 3 shows the relationship between physical activity and all-cause mortality risk according to physical activity intensity using a restricted cubic spline curve. A non-linear relationship between LPA and mortality risk showed a continuous decrease in mortality risk until 360 MET-min/week (120 min/week: HR 0.63, 95% CI 0.57–0.71) and a plateau. The relationship between MVPA and mortality risk showed a continuous decrease in mortality risk until 528 MET-min/week or 132 min/week. For the same exercise duration, MVPA was associated with lower all-cause mortality than LPA.

### Subgroup Analysis

The HR for all-cause mortality according to physical activity dose in different subgroups in those who did not perform any activity beyond LPA is presented in Supplementary Figure 5 and shows a consistent decrease in risk regardless of subgroups except in subjects with obesity and COPD. The HR for all-cause mortality according to physical activity intensity in different subgroups in the overall population is presented in Supplementary Figure 6.

## Discussion

In this study, 46,347 (79.2%) older participants did not perform any activity beyond LPA. Second, compared with those in the totally sedentary group, older adults performing LPA alone had a reduced risk of all-cause mortality, CV mortality, and non-CV mortality. Third, this finding was consistently observed regardless of the comorbidities. Finally, the risk of all-cause mortality was continuously reduced by LPA alone until 360 MET-min/week and reached a plateau. These results suggest that light physical activity alone can be beneficial in reducing mortality in older adults.

### Physical Activity and Mortality in Older Adults

It is estimated that >60% of older people could not achieve 150 min per week of MVPA (6). In this study, 82.7% (55,616/68,500) of older adults were not able to achieve guideline-recommended exercise, suggesting that it may have been too demanding for them. Moreover, 79.2% of the older adults did not have physical activity beyond LPA.

Recent studies reported that the effect of LPA might be beneficial in older adults (7, 21, 22). Previous guidelines recommended MPA or VPA alone in elderly adults, although recent studies showed that LPA was also associated with improving cardio-metabolic health and reducing mortality risk (23). Another study showed that sedentary behavior was associated with negative health effects (24). Therefore, recently published WHO 2020 Guidelines on physical activity recommended replacing sedentary time with physical activity of any intensity, including LPA (3). The latest International Exercise Recommendations in Older Adults suggested that aerobic exercises including walking, stair climbing, stationary cycling, dancing, and aquatic exercise may start with a duration of 5–10 min and progress to 15–30 min with appropriate intensity using heart rate and/or perceived exertional scales (25). However, recent studies on LPA and mortality had a small number of participants and did not show consistent results (26, 27).

This study showed that all-cause mortality can be reduced even with LPA alone in older adults. Manson et al. (28) reported that both walking and vigorous exercise are associated with reduced cardiovascular events among 73,743 postmenopausal women aged 50–79 years. According to Saint-Maurice et al.’s study (29), based on a representative sample of US adults (mean age, 56.8 years), daily step count was significantly associated with all-cause mortality. However, higher step intensity was not associated with lower mortality after adjusting for the total number of steps per day.

### The Dose-Response Relationship Between Physical Activity and Mortality

We found the dose-response relationship between physical activity and mortality by calculating the total amount of physical activity as a continuous variable. The positive effect of increased physical activity on mortality started at a low dose of total physical activity. These results imply that even a low dose of physical activity, rather than sedentary behavior, could reduce the mortality risk.

The reduction in the risk of all-cause mortality was decreased in both LPA and MVPA with a further increase in the amount of exercise. The risk of all-cause death was reduced by 37% at 360 MET-min/week and by 49% at 528 MET-min/week in the LPA only and MVPA only groups, respectively. This finding suggests that the reduction in all-cause mortality is greater with more intensive exercise. Wang et al. (30) suggested that participants with a higher proportion of VPA to total physical activity had a lower mortality.

The physical activity for older adults should be individualized according to their biological age, comorbidity, safety, and functional capacity (31). Nauman et al. (32) suggested that personalized metric for physical activity using individual’s sex, age, and heart rates was useful for quantifying the amount of PA needed to produce significant health benefits. Recommendation from the American College of Sports Medicine and American Heart Association (ACSM/AHA) define aerobic intensity for healthy adults in absolute terms, e.g., moderate-intensity comprises 3.0–6.0 MET activities. The older adult recommendation from ACSM/AHA defines exercise intensity as relative to fitness (33). The range of 30–60 min of light-intensity activity a day could be sufficient in older adults with functional limitations.

### Limitation

This study had some limitations. First, such studies using administrative databases could be susceptible to errors arising from coding inaccuracies. To minimize this issue, we used the same definitions that we had already validated in previous studies using the Korean NHIS cohort (10, 12–20). Second, it relied on self-reported data on physical activity, as collected at baseline. The answer at the time of questionnaire completion may not represent the actual physical activity status throughout life. Furthermore, behavioral changes that occurred during the follow-up period could not be assessed in this study. Also, it might be inappropriate to assign MET levels for the respective intensity groupings without knowing the cardiorespiratory fitness levels of the participants. This is because a proportion of the older adults likely had low fitness levels which would have made it difficult to perform activity in between 4 and 8 and above 8 METs. Despite these limitations, this study has strength in that it included a large number of older adults who had physical activity data. We assessed the dose-response relationship between physical activity and mortality and focused on the effect of LPA on the incidence of mortality. Various sensitivity analyses showed consistent results, which supports our main result.

## Conclusion

The analyses of the Korean NHIS-Senior database showed a reduced mortality risk in individuals with LPA than in those with totally sedentary behavior. The mortality risk was reduced in a dose-dependent manner even less than the recommended dose of physical activity. Replacing sedentary time with any activity, even low doses of LPA could play a role in reducing the mortality risk in inactive older adults.

## Data Availability Statement

The original contributions presented in the study are included in the article/Supplementary Material, further inquiries can be directed to the corresponding author.

## Ethics Statement

The studies involving human participants were reviewed and approved by Institutional Review Board of the Yonsei University Health System (4-2021-0850). Written informed consent for participation was not required for this study in accordance with the national legislation and the institutional requirements.

## Author Contributions

JK and BJ designed the study. P-SY, B-EP, TSK, and S-HL assisted with data acquisition and interpretation. SC, S-YL, and YHC performed statistical analyses. DK and M-YL contributed to the discussion. JK, DK, and BJ drafted the manuscript. P-SY and BJ revised the manuscript. All authors read and approved the final manuscript.

## Conflict of Interest

BJ has served as a Speaker for Bayer, BMS/Pfizer, Medtronic, and Daiichi-Sankyo and received research funds from Medtronic and Abbott. The remaining authors declare that the research was conducted in the absence of any commercial or financial relationships that could be construed as a potential conflict of interest.

## Publisher’s Note

All claims expressed in this article are solely those of the authors and do not necessarily represent those of their affiliated organizations, or those of the publisher, the editors and the reviewers. Any product that may be evaluated in this article, or claim that may be made by its manufacturer, is not guaranteed or endorsed by the publisher.

## Abbreviations

ACSM/AHA: American College of Sports Medicine and American Heart Association
CI: confidence interval
CV: cardiovascular
HR: hazard ratio
ICD-10: The International Classification of Disease-10th revision
LPA: light-intensity physical activity
MET: metabolic equivalent task
MPA: moderate-intensity physical activity
MVPA: moderate- to vigorous-intensity physical activity
NHIS: The National Health Insurance Service of Korea
VPA: vigorous-intensity physical activity.
