# Supplementary material for: Association of Light-Intensity Physical Activity With Mortality in the Older Population: A Nationwide Cohort Study
Source: Front Cardiovasc Med. 2022 Apr 22;9:859277. doi: 10.3389/fcvm.2022.859277 (PMC9072650; doi:10.3389/fcvm.2022.859277)
Supplement: Supplementary file 1 [file Data_Sheet_1.pdf]

Supplementary Material

Supplementary Table 1. Definitions and ICD-10 codes used for defining the comorbidities and clinical outcomes.

Supplementary Figure 1. Distribution of dose of exercise according to intensity of exercise.

Supplementary Figure 2. Cumulative incidence curves of all cause death and CV cause death in overall population.

Supplementary Figure 3. Cumulative incidence curves of all cause death and CV cause death in those who did not perform any activity beyond LPA.

Supplementary Figure 4. Hazard ratio with 95% confidence intervals for all cause death, CV cause death, and non-CV-cause death in those who did not perform any activity beyond LPA, according to energy expenditure (MET-min/week).

Supplementary Figure 5. Hazard ratio with 95% confidence intervals for all-cause death, CV-cause death, and non-CV-death in patients with moderate- to vigorous-intensity physical activity, according to energy expenditure (MET-min/week).

Supplementary Figure 6. Hazard ratio for all-cause mortality in different subgroups in overall population.

Supplementary Table 1. Definitions and ICD-10 codes used for defining the comorbidities and clinical outcomes.

	Definitions	ICD-10 codes or conditions
**Comorbidities**		
Chronic kidney disease	Defined from eGFR or diagnosis* (if laboratory value was not available, diagnosis code was used)	eGFR <60mL/min per 1.73 m^2<br>ICD-10: N18, N19
Chronic Liver disease	Defined from diagnosis of chronic liver disease, cirrhosis, and hepatitis	ICD-10: B18, K70, K71, K72, K73, K74, K76.1
Chronic obstructive pulmonary disease	Defined from diagnosis* plus treatment	ICD-10: J42, J43(except J43.0), J44<br>Treatment: SABA, SAMA, LABA, LAMA, ICS, ICS+LABA, or methylxanthine (>1 months).
Diabetes mellitus	Defined from diagnosis* plus treatment	ICD-10: E10, E11, E12, E13, E14<br>Treatment: all kinds of oral antidiabetics and insulin.
Dyslipidemia	Defined from diagnosis*	ICD-10: E78
End-stage renal disease	Defined from national registry for severe illness.	Patients with end-stage renal disease undergoing chronic dialysis or received a kidney transplant.
Hypertension	Defined from diagnosis*	ICD-10: I10, I11, I12, I13, I15 and antihypertensive medication
Malignancy	Defined from diagnoses of cancer (non-benign)	ICD-10: C00-C97
**Clinical outcomes**		
All-cause death	Data related to death were confirmed at the National Population Register of the Korea National Statistical Office, where deaths are centrally registered based on the death certificate.	The cause of death was determined based on the ICD-10 code written on the death certificate.
Cardiovascular death	Defined from related death	ICD-10: I00-I78

*To ensure accuracy, comorbidities were established based on one inpatient or two outpatient records of ICD-10 codes in the database.

eGFR, estimated glomerular filtration rate; ICD-10, International Classification of Diseases-10th Revison

Supplementary Figure 1. Distribution of dose of exercise according to intensity of exercise.

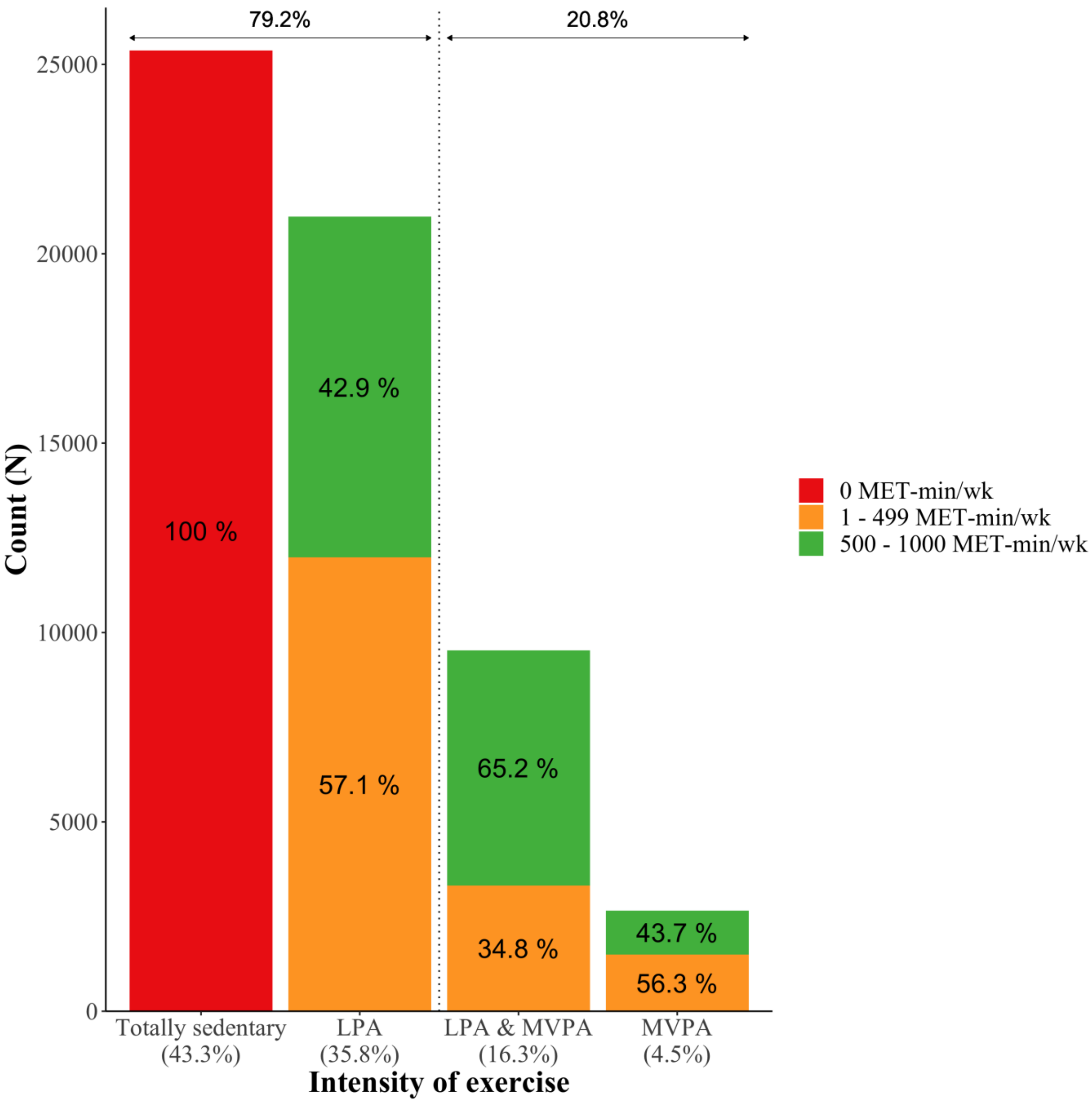

LPA, light-intensity physical activity; MVPA, moderate- to vigorous-intensity physical activity; MET, metabolic equivalent of task.

Supplementary Figure 2. Cumulative incidence curves of all-cause death, CV-cause death, and non-CV cause death in overall population.

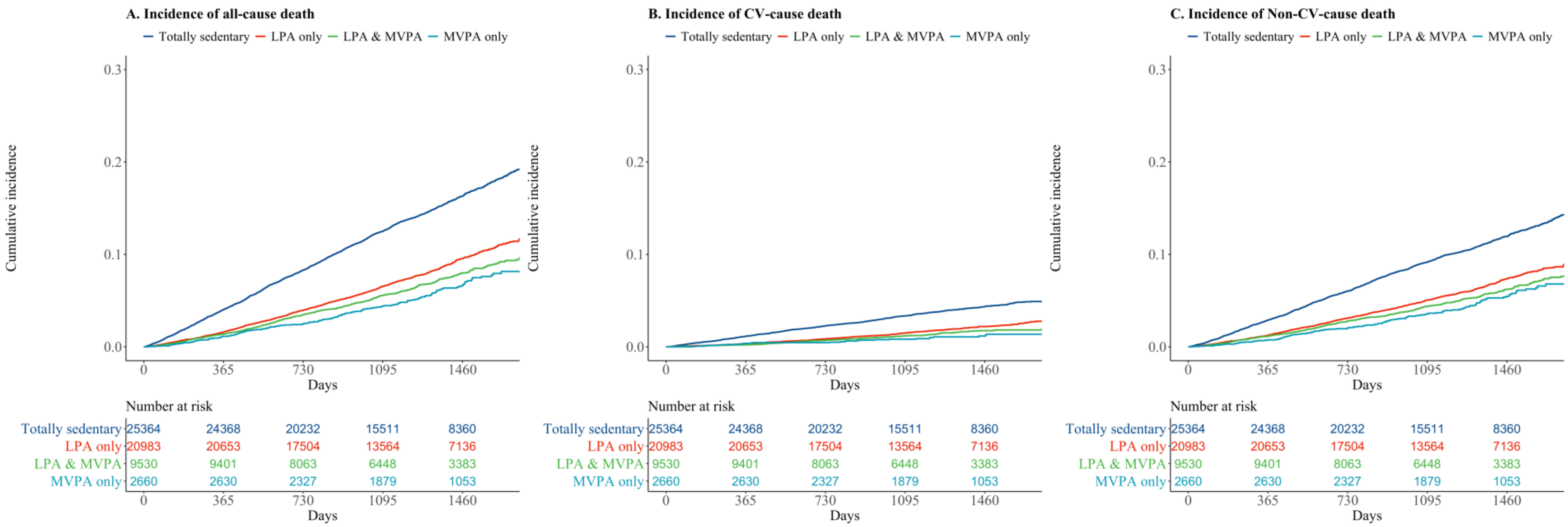

LPA, light-intensity physical activity; MVPA, moderate- to vigorous-intensity physical activity; MET, metabolic equivalent of task.

Supplementary Figure 3. Cumulative incidence curves of all-cause death, CV-cause death, and non-CV cause death in those who did not perform any activity beyond light-intensity physical activity.

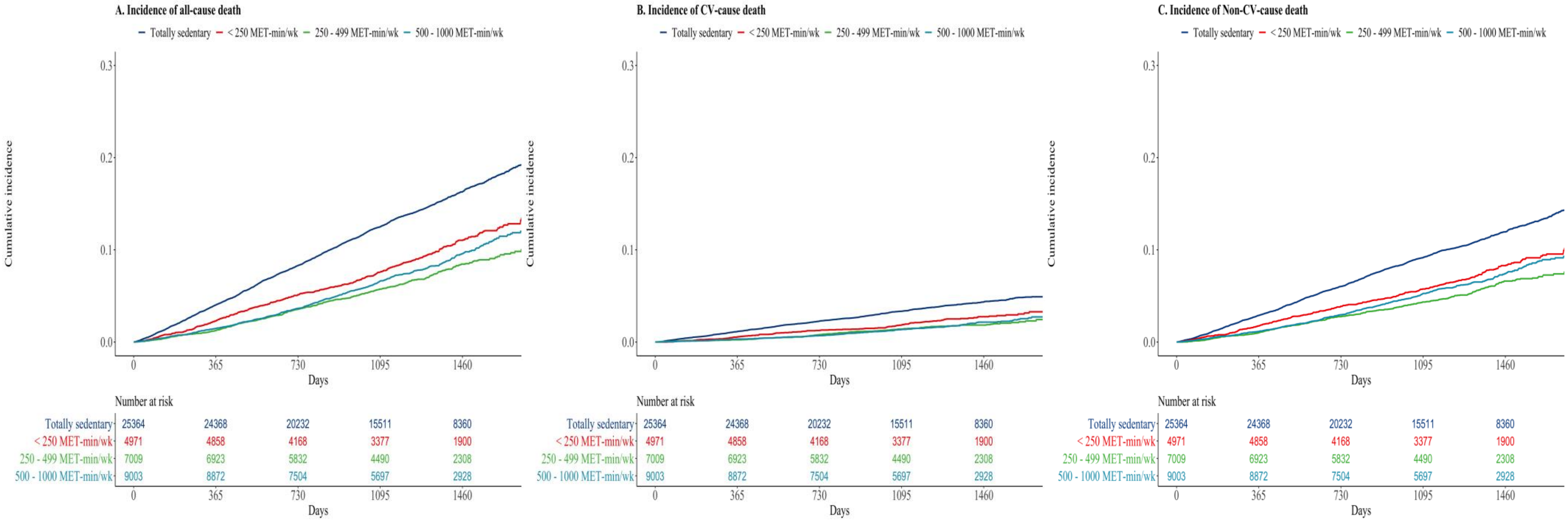

MET, metabolic equivalent of task.

Supplementary Figure 4. Hazard ratio with 95% confidence intervals for all-cause death, CV-cause death, and non-CV-death in patients with moderate- to vigorous-intensity physical activity, according to energy expenditure (MET-min/week). The bars denote incidence rates, the dots denote hazard ratios, and the whiskers denote 95% confidence intervals computed by Cox proportional hazards models.

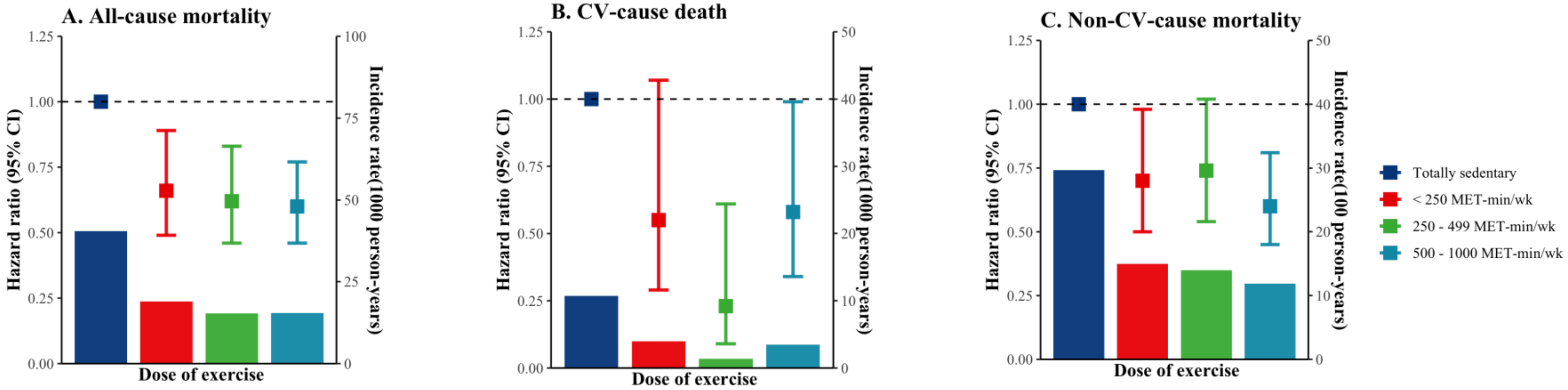

MET, metabolic equivalent of task.

Supplementary Figure 5. Hazard ratio for all-cause mortality in different subgroups in those who did not perform any activity beyond light-intensity physical activity. Boxes indicate the hazard ratio, limit lines indicate the 95% confidence interval, and the vertical line (at hazard ratio 1) indicates no difference in the hazard.

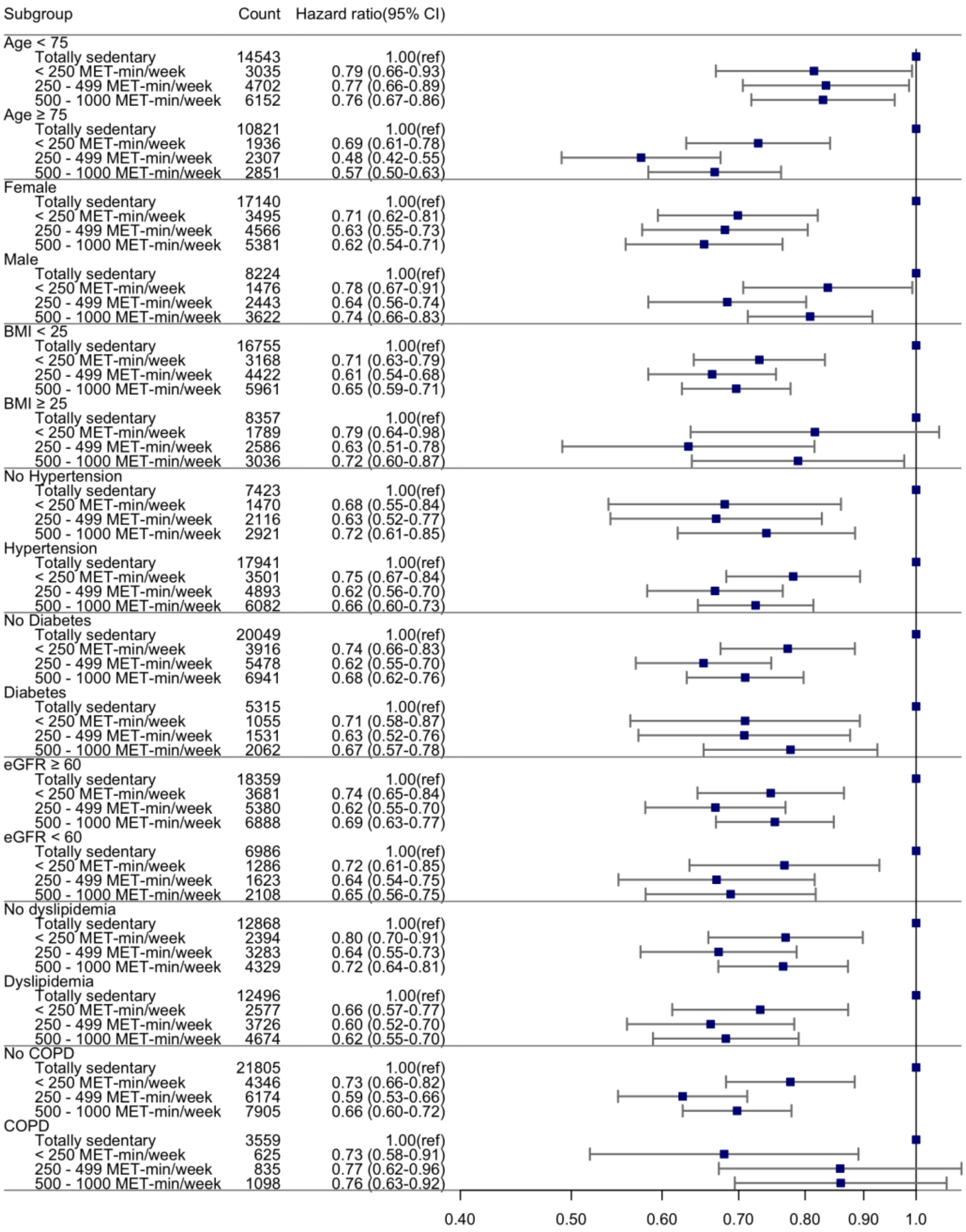

MET, metabolic equivalent of task.

Supplementary Figure 6. Hazard ratio for all-cause mortality in different subgroups in overall population. Boxes indicate the hazard ratio, limit lines indicate the 95% confidence interval, and the vertical line (at hazard ratio 1) indicates no difference in the hazard.

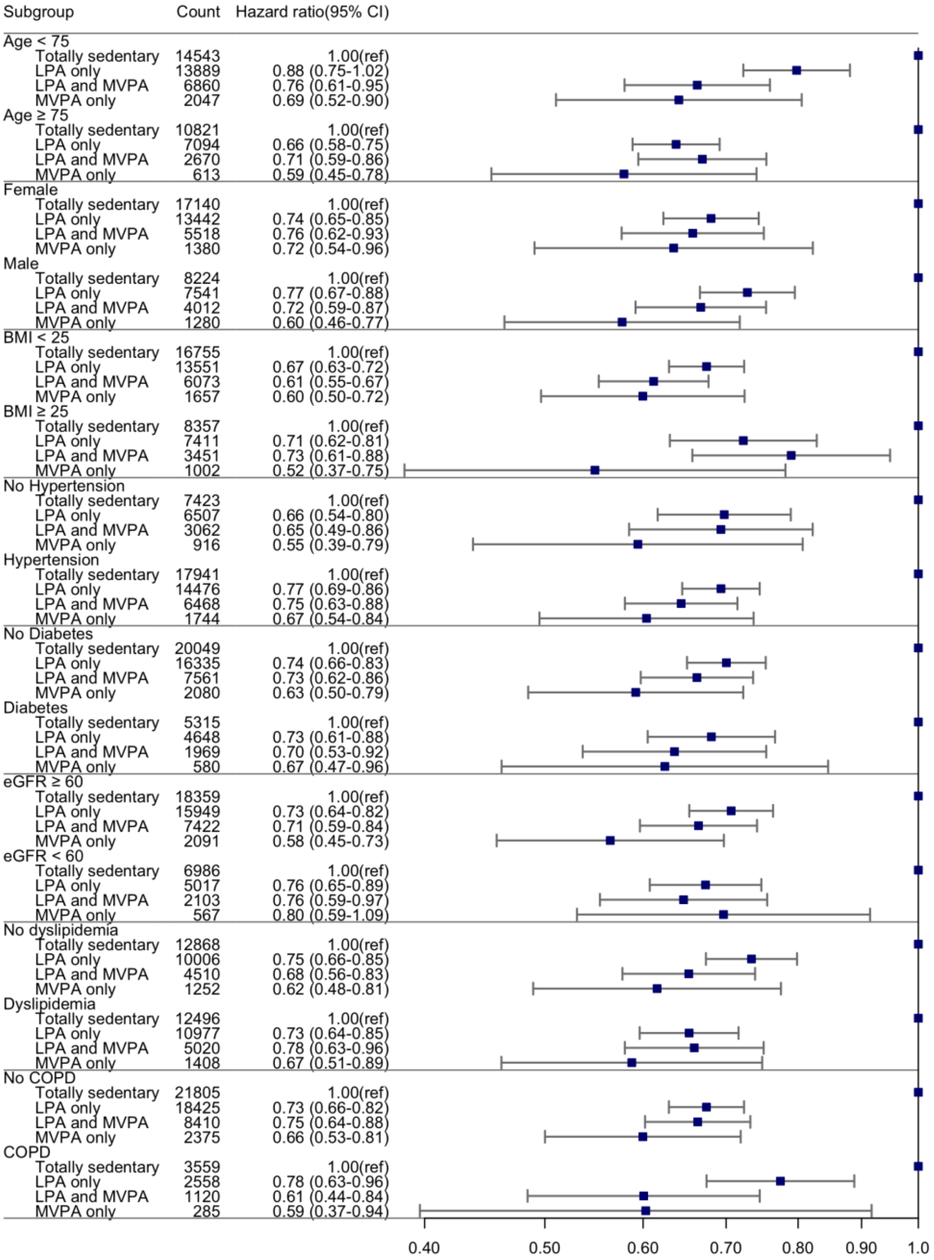

LPA, light intensity physical activity; MVPA, moderate to vigorous intensity physical activity; MET, metabolic equivalent of task.
